# Minimizing Acute Kidney Injury in Pediatric Cardiac Surgery: Incidence, Early Detection, and Preemptive Measures

**DOI:** 10.7759/cureus.72916

**Published:** 2024-11-03

**Authors:** Vipul Sharma, Harika Atluri

**Affiliations:** 1 Anaesthesiology, Dr. D Y Patil Medical College, Hospital and Research Centre, Dr. D Y Patil Vidyapeeth (Deemed to be University), Pune, IND

**Keywords:** : acute kidney injury, cardiopulmonary bypass, cystatin c, hematocrit, neutrophil gelatinase-associated lipocalin (ngal), pediatric cardiac surgery, serum creatinine, vasopressin

## Abstract

Background

Acute kidney injury (AKI) poses a significant challenge in pediatric cardiac surgery, having a profound impact on patient morbidity and mortality. This study aims to determine the incidence of AKI, explore novel biomarkers for early detection, assess potential risk factors along with preemptive strategies to minimize its incidence and compare the results with similar studies that did not use these interventions.

Methods

This prospective observational cohort study, conducted from October 2022 to June 2024 at a tertiary care center, involved 44 pediatric patients, aged three months to 15 years, undergoing cardiac surgery. Kidney function was assessed through preoperative and postoperative measurements of serum creatinine, urine output, blood urea, and newer biomarkers such as cystatin C and urine neutrophil gelatinase-associated lipocalin (NGAL). AKI was defined and classified using the Acute Kidney Injury Network (AKIN) criteria, based on increases in serum creatinine or reductions in urine output within the first three days post surgery. To reduce the risk of AKI, a low-dose vasopressin infusion and blood transfusion were administered to maintain renal perfusion and optimal hematocrit levels. The incidence of AKI was calculated and compared with other studies that did not utilize these strategies

Results

AKI occurred in 31.8% (n=14) of the pediatric patients undergoing cardiac surgery. To reduce the risk of AKI, preemptive low-dose vasopressin was used as a preventive strategy. Patients who developed AKI exhibited significant elevations in serum creatinine, blood urea, and cystatin C, with postoperative NGAL levels exceeding 50 ng/ml. The study found a strong correlation between lower intraoperative hematocrit levels (<30%) and a higher incidence of AKI (100% vs. 6.2%, p<0.001).

Conclusions

Effective management of intraoperative hematocrit levels and the preemptive use of vasopressin are promising strategies for reducing AKI risk by optimizing renal perfusion and function during cardiac surgery. Early detection through biomarkers like cystatin C and NGAL offers the potential for timely intervention and better patient outcomes. These findings contribute to improving risk assessment and perioperative management in pediatric patients vulnerable to AKI.

## Introduction

Acute kidney injury (AKI) is a significant complication in pediatric cardiac surgery, marked by a rapid decline in the glomerular filtration rate (GFR) and the accumulation of nitrogenous waste products like urea and creatinine. Its impact on both short- and long-term outcomes is profound, leading to increased morbidity and mortality rates. The incidence of AKI in this context was estimated at 5-8% in the past, focusing mainly on later stages [1,2]. However, with a contemporary definition allowing for earlier identification, the incidence of pediatric cardiac surgery AKI (CS-AKI) now ranges from 9.6% to 42% in children, with even higher rates in infants (52%) and neonates (64%) undergoing biventricular cardiac repair [3-6]. Notably, children under two years of age face a heightened risk of AKI [4].

The etiology of CS-AKI is multifaceted, encompassing pre-renal, intrinsic renal, and post-renal causes. Cardiac surgery often elevates peripheral vascular resistance and disrupts microcirculation, leading to ischemia-reperfusion injury and tissue edema [7]. Furthermore, the associated ischemia-reperfusion injury and cardiopulmonary bypass exposure trigger systemic inflammation, promoting the upregulation of pro-inflammatory factors and infiltration of immune cells into the renal parenchyma, ultimately resulting in fibrosis [8]. Elevated postoperative levels of inflammatory cytokines correlate with the incidence of AKI and increased mortality rates [9].

The ramifications of CS-AKI extend beyond renal impairment, affecting various facets of patient care. Increased need for inotropic support, need for dialysis, susceptibility to infections, prolonged mechanical ventilation, ventilator-associated pneumonia, and extended stays in the intensive care unit (ICU) and hospital characterize its clinical course. Mortality in CS-AKI stems from a plethora of factors, including fluid overload-related lung complications, increased infection risk due to mechanical ventilation, and the cardio-renal interplay mediated by cytokines and chemokines [10-12].

With this background, we aimed to evaluate the increase in serum creatinine and the decrease in urine output post pediatric cardiac surgery in this study. Additionally, we aimed to assess AKI using early kidney injury biomarkers, i.e., neutrophil gelatinase-associated lipocalin (NGAL) and cystatin C. We evaluated the association between AKI and intraoperative hematocrit levels and explored the effect of preemptive low-dose vasopressin infusion.

## Materials and methods

Study design

This prospective observational cohort study was conducted in the Cardiothoracic and Vascular Surgery Department at Dr. D. Y. Patil Medical College, Hospital and Research Centre, a leading tertiary care facility in Pune. The study was conducted from October 2022 to June 2024, with the first year focused on data collection and the subsequent months on data processing and analysis. Before initiation, the study was approved by Dr.D.Y.Patil Medical College, Hospital and Research Centre Institutional Ethics Sub-Committee (approval number: IESC/PGS/2022/156). A pre-anesthetic assessment was conducted for all patients and informed written consent was obtained from the parents or legal guardians of the pediatric patients, who provided comprehensive information.

The study included children aged between three months and 15 years, regardless of sex, who were scheduled for cardiac surgery. Eligible participants were required to have an American Society of Anesthesiologists (ASA) physical status classification of II, III, or IV, and their weight needed to be within the normal range for their age and sex. Additionally, informed written consent had to be provided by the parents or legal guardians of the patients. Conversely, patients older than 15 years, those with pre-existing renal disorders, or individuals presenting with sepsis were excluded from the study. Furthermore, any potential participants who did not provide consent were also excluded from the study.

Sample size calculation

The sample size was calculated using WinPepi version 11.65, based on a previous study by Park et al. reporting a 41.8% incidence of pediatric CS-AKI, with a power of 80% and a 95% confidence interval (CI), yielding a required sample size of 42 [13].

Protocol

The preoperative evaluation for each participant in this study was extensive and detailed. Initially, a series of baseline investigations were conducted to establish the patient's health status before undergoing surgery. These included chest X-ray, electrocardiogram (ECG), and a two-dimensional echocardiogram (2D Echo) to assess the condition of the lungs and heart in detail. Additionally, routine surgical blood workups were performed, which encompassed a comprehensive analysis of blood parameters and urine analysis to detect any underlying renal or systemic issues. Key biochemical markers such as serum creatinine, blood urea, and cystatin C levels were also measured to assess renal function and establish a baseline for postoperative comparison.

On the day of surgery, the patient’s preoperative preparation included an intramuscular (IM) or intravenous (IV) injection of vitamin K at a dose of 0.2 mg/kg to support blood clotting. Sildenafil syrup, at a dose of 0.25-0.5 mg/kg, was administered to manage pulmonary hypertension, while Triclofos syrup (75 mg/kg) was given 45 minutes prior to surgery by the doctor in the ward with all monitors attached to promote sedation. For further sedation and analgesia, nasal ketamine (7 mg/kg) combined with midazolam (0.4 mg/kg) was administered, split between both nostrils while the patient remained on the mother’s lap. Twenty minutes after nasal premedication, the patient was transferred to the operating room (OR).

Induction of anesthesia began with bag-mask ventilation using 60% fraction of inspired oxygen (FiO2), carefully avoiding 100% FiO2 to minimize the risk of oxygen toxicity. Following a standard protocol, anesthesia induction, intubation, and maintenance were customized to each patient’s requirements. Maintenance fluids were precisely calculated, consisting of 10% dextrose in N/3 (3% NaCl) saline with added calcium gluconate, potassium chloride (KCl), midazolam, and magnesium sulfate (MgSO4), all delivered at 1 ml/kg/hour, ensuring optimal fluid and electrolyte management throughout the procedure.

A preemptive low-dose vasopressin infusion was administered immediately following anesthesia induction at 0.0003 units/kg/minute and continued throughout the intraoperative period. Blood transfusions were administered at a rate of 4 ml/kg/hour as necessary to maintain optimal hematocrit (>30%) to enhance renal perfusion and support kidney function. During the cardiopulmonary bypass phase, intraoperative arterial blood gas samples were collected to monitor and manage the metabolic status.

Throughout both the intraoperative and postoperative periods, continuous monitoring was essential. Urine and blood samples were systematically collected, and we measured the biomarkers at multiple intervals, with serum creatinine and urine output being measured at 24 hours and three days postoperative, cystatin C at six hours postoperatively, and NGAL at two hours postoperatively to track fluctuations and compare with the preoperative values. Hematocrit levels and basic urine analysis were also conducted to evaluate renal function and detect early signs of AKI. Post-extubation care involved the provision of high-flow nasal air and supplemental oxygen to support respiratory recovery.

AKI was defined and classified according to the AKIN criteria, with AKI identified based on postoperative increases in serum creatinine or reductions in urine output within the first three days after surgery. The incidence of AKI was calculated as the proportion of patients who developed AKI out of the total number of patients included in the study. Specifically, the number of patients meeting the AKIN criteria for AKI was divided by the total sample size (44 patients), and the resulting percentage was reported as the incidence rate. To contextualize our findings, the incidence of AKI in our study was compared with previously published studies that did not implement the use of preemptive vasopressin or transfusion strategies. This comparison highlights the potential effectiveness of preemptive vasopressin infusion and transfusion management in reducing the incidence of AKI, as our study observed a comparatively lower incidence rate. Such comparisons help to demonstrate the impact of the strategies we employed, relative to studies that did not use similar preemptive measures.

Data was recorded on standardized sheets, transferred to Excel (Microsoft Corporation, Redmond, Washington, United States), and cross-checked by two researchers for accuracy. After ensuring consistency, IBM SPSS Statistics for Windows, Version 26.0 (Released 2019; IBM Corp., Armonk, New York, United States) was used for analysis, considering p-values <0.05 as significant. To maintain patient confidentiality, data was securely stored on password-protected systems with encrypted backups. As per institutional policy, the data will be preserved for at least five years.

## Results

**Figure 1:**
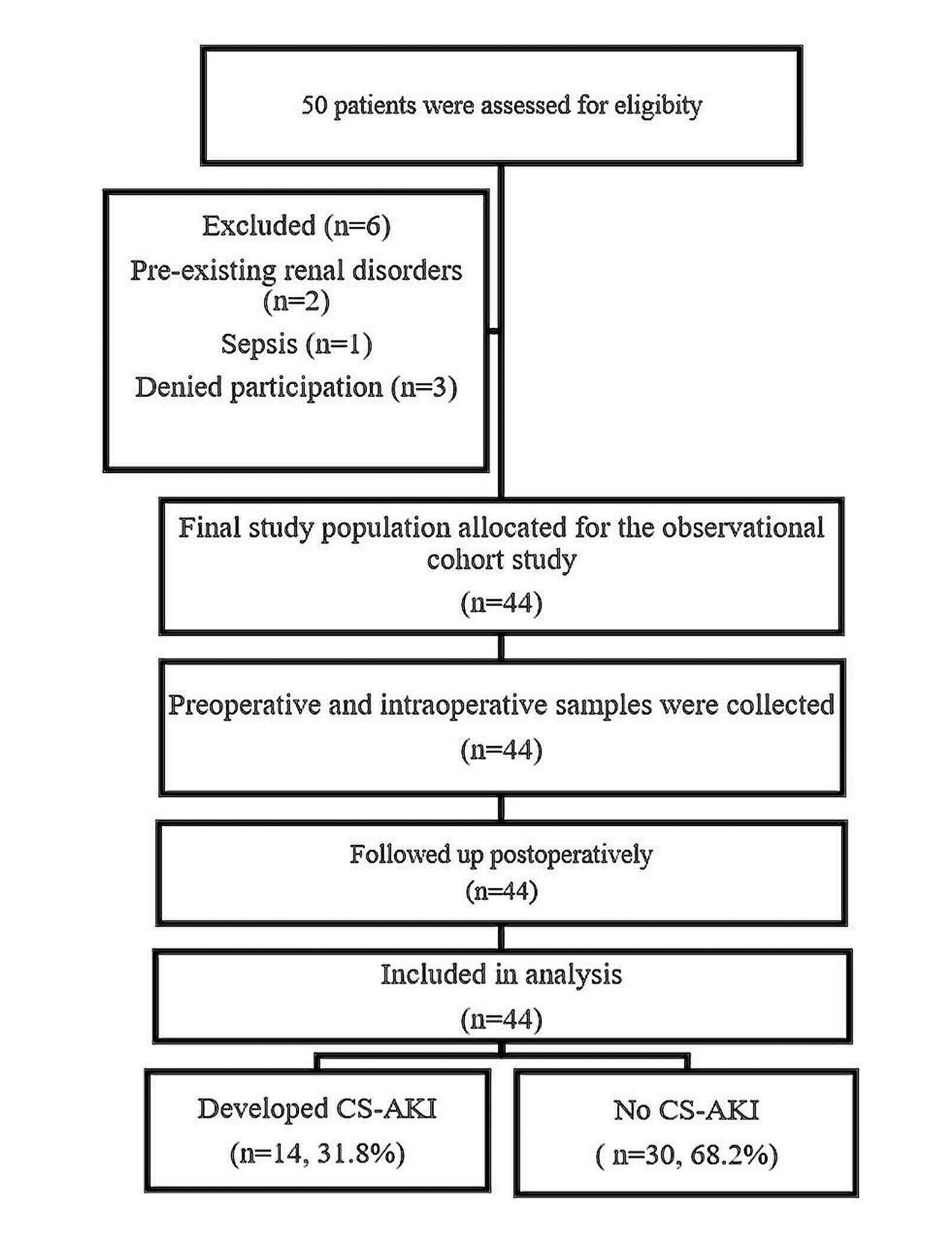
STROBE chart of Observational Study STROBE: Strengthening the Reporting of Observational Studies in Epidemiology; CS-AKI: cardiac surgery-associated acute kidney injury

In this observational cohort study, we initially assessed 50 pediatric patients for eligibility. Of these, six were excluded, leaving 44 patients who completed the study. All enrolled participants were included in the analysis and underwent thorough assessment and evaluation throughout the study (Figure 1).

Understanding the fundamental demographic and clinical features of study participants is vital for interpreting the study cohort and evaluating how various factors might affect the incidence of AKI. A comprehensive analysis of factors including age, gender, weight, height, cardiovascular findings, and diagnoses (Table 1).

**Table 1:** Demographic distribution of the pediatric study population. CVS: cardiovascular system; ASD: atrial septal defect; VSD: ventricular septal defect; TOF: tetralogy of Fallot; TAPVC: total anomalous pulmonary venous connection

Demographics		Frequency	Percentage
Age (years)	≤1	29	65.9%
	1-5	7	15.9%
	6-10	8	18.2%
Gender	Male	24	54.5%
	Female	20	45.5%
Weight (Kg)	1-5	20	45.5%
	6-10	11	25.0%
	11-15	8	18.2%
	16-20	2	4.5%
	21-25	3	6.8%
Height (cm)	≤ 60	13	29.5%
	60-90	19	43.2%
	>90	12	27.3%
CVS	Murmur +	17	38.6%
	NAD	27	61.4%
Diagnosis	ASD	9	20.5
	VSD	21	47.7
	ASD+VSD	1	2.3
	TOF	6	13.6
	TAPVC	7	15.9

The AKI was classified according to the AKIN criteria, which included increased serum creatinine levels ≥ 0.3 mg/dL and decreased urine output to less than 0.5 mL/kg/hour for six hours within the first three days following surgery (Table 2). In our study, 14 out of 44 patients developed AKI, resulting in an incidence rate of 31.8%.

**Table 2:** Classification of patients into AKI and non-AKI groups based on AKIN criteria AKIN: Acute Kidney Injury Network; AKI: acute kidney injury

Parameters (AKIN Criteria)	AKI Group	Non-AKI Group
Serum Creatinine (mg/dL)	0.8-1.6	0.4-0.7
Urine Output (mL/kg/hr)	0.1-0.5	1.2-1.7
Number of Patients	14	30

The mean blood urea levels significantly increased on postoperative day three compared to preoperative levels (p=0.008). This increase in blood urea levels is an indicator of renal dysfunction, as the kidneys are unable to effectively eliminate nitrogenous waste products like urea. The mean serum creatinine levels significantly increased on postoperative day one and day three compared to preoperative levels (p<0.001). Serum creatinine is a commonly used marker for assessing kidney function, and its elevation suggests a decline in glomerular filtration rate and renal impairment. The mean cystatin C levels also significantly increased at six hours compared to preoperative levels (p<0.001) (Table 3). 

**Table 3:** Blood urea, serum creatinine, and cystatin C at different time points in the study The data are presented as the mean ± SD and 95% CI (mean ± CI value) for each measurement and p-value at specified time intervals. An asterisk (*) indicates p-values that represent statistically significant differences compared to preoperative levels, where p-values less than 0.05. POD: postoperative day

Parameter	Timepoint	Mean±SD	95% CI (Mean ± CI Value)	P value
Blood urea	Preoperative	32.41±4.55	32.41 ± 1.56	-
	POD 1	41.73±2.39	41.73 ± 0.82	0.056
	POD 3	47.86±3.9	47.86 ± 1.34	0.008*
Serum Creatinine	Preoperative	0.402±0.098	0.402 ± 0.033	-
	POD 1	0.542±0.188	0.542 ± 0.065	<0.001
	POD 3	0.589±0.255	0.589 ± 0.088	<0.001
Cystatin C	Preoperative	0.495±0.083	0.495 ± 0.028	-
	At 6 hours	0.136±0.009	0.136 ± 0.003	<0.001*

In this study, mean serum creatinine and blood urea levels were significantly elevated in some patients on postoperative days one and three, aligning with the AKIN criteria for diagnosing AKI. This led to the classification of patients into AKI and non-AKI groups. Moreover, patients identified with AKI based on increased serum creatinine also showed a significant rise in serum cystatin C as early as six hours post surgery (p<0.001). These findings suggest that cystatin C could serve as an early biomarker for AKI, offering earlier detection and identification of at-risk patients before notable renal dysfunction occurs, compared to serum creatinine (Table 4). The increase in cystatin C levels may indicate early renal injury or impaired glomerular filtration.

**Table 4:** Association between AKI and serum creatinine and cystatin-C The data are presented as the mean ± SD and 95% CI (mean ± CI value) for each measurement and p value at specified time intervals. An asterisk (*) indicates p-values that represent statistically significant differences compared to preoperative levels, where p-values are less than 0.05. AKI: acute kidney injury; POD: postoperative day

Parameter	Timepoint	AKI Mean ±SD (Mean ± CI Value)		P value
		Present	Absent	
Serum Creatinine	Preoperative	0.409 ±0.040 (0.409 ± 0.014)	0.398 ±0.011 (0.398 ± 0.004)	0.744
	POD 1	0.791 ±0.026 (0.791 ± 0.009)	0.425 ±0.012 (0.425 ± 0.004)	<0.001*
	POD 3	0.935 ±0.030 (0.935 ± 0.010)	0.428 ±0.0145 (0.428 ± 0.005)	<0.001*
Cystatin C	Preoperative	0.708 ±0.154 (0.708 ± 0.199)	0.396 ±0.094 (0.396 ± 0.104)	0.080
	At 6 hours	0.211±0.012 (0.211 ± 0.004)	0.102 ±0.0045 (0.102 ± 0.007)	<0.001*

Patients with elevated NGAL levels (>50 ng/mL) two hours after surgery had significantly higher mean postoperative serum creatinine levels (p<0.001) compared to those with lower NGAL levels (<50 ng/mL), indicating NGAL's potential as an early marker for AKI due to its strong association with subsequent creatinine increases. Furthermore, mean cystatin C levels measured six hours postoperatively were significantly higher in patients with elevated NGAL levels at two hours (p<0.001), reinforcing the complementary roles of NGAL and cystatin C in the early detection of AKI (Table 5).

**Table 5:** Association between NGAL and serum creatinine and cystatin The data are presented as the mean ± SD and 95% CI (Mean ± CI Value) for each measurement and p-value at specified time intervals. An asterisk (*) denotes statistically significant differences compared to preoperative levels, with significance defined as p-values less than 0.05. NGAL: neutrophil gelatinase-associated lipocalin; POD: postoperative day

Parameter	Timepoint	NAGL Mean ±SD (Mean ± CI Value)		P value
		<50 (n=30)	>50 (n=14)	
Serum Creatinine	Preoperative	0.398 ±0.065 (0.398 ± 0.022)	0.409 ±0.150 (0.409 ± 0.051)	0.744
	POD 1	0.425 ±0.066 (0.425 ± 0.022)	0.791 ±0.099 (0.791 ± 0.034)	<0.001*
	POD 3	0.428 ±0.079 (0.428 ± 0.028)	0.935 ±0.114 (0.935 ± 0.039)	<0.001*
Serum Cystatin C	Preoperative	0.396 ±0.516 (0.396 ± 0.198)	0.708 ±0.579 (0.708 ± 0.199)	0.080
	At 6 hours	0.102 ±0.024 (0.102 ± 0.009)	0.211 ±0.045 (0.211 ± 0.016)	<0.001*

All patients with a low intraoperative hematocrit level <30 developed AKI (100%), while only 6.2% of patients with intraoperative hematocrit level >30 developed AKI (p<0.001). This finding suggests that maintaining adequate hematocrit levels during cardiac surgery is crucial for preserving renal perfusion and preventing AKI (Table 6).

**Table 6:** Association between lowest intraoperative hematocrit and AKI The data are presented as percentages, representing the proportion of patients within each AKI status group. An asterisk (*) denotes statistically significant differences compared to preoperative levels, with significance defined as p-values less than 0.05. AKI: acute kidney injury

AKI	Lowest intraoperative hematocrit, n (%)		P Value
	<30	>30	
Yes, n (%)	12 (100%)	2 (6.2%)	<0.001*
No, n (%)	0 (0%)	30 (93.8%)	

## Discussion

The incidence of AKI in our study was 31.8%, aligning with previous reports that range from 4% to 76%, with differences likely due to variations in patient populations, surgical techniques, and the AKI criteria used. We employed the AKIN criteria, with a postoperative follow-up period of three days, providing a standardized basis for comparison. Other criteria, such as KDIGO (Kidney Disease: Improving Global Outcomes) and pRIFLE (Pediatric Risk, Injury, Failure, Loss, End Stage Renal Disease), can also be effective in diagnosing AKI in pediatric patients. Typically, CS-AKI is monitored up to seven days after surgery, with the highest risk occurring within the first 48-72 hours, largely due to the effects of cardiopulmonary bypass. The AKIN criteria focus on detecting AKI within three days, while KDIGO extends the monitoring window to seven days, capturing both early and delayed AKI. Studies suggest that the AKIN system is more specific for identifying high-risk patients, while pRIFLE is more sensitive for early AKI detection [8].

Our study found a significant increase in serum creatinine and blood urea levels, and according to AKIN criteria, they were classified into AKI [14]. While increases in serum creatinine and blood urea levels indicate renal dysfunction, they have limitations such as delayed rise after renal insult and influence by non-renal factors [15]. To address this, we explored alternative biomarkers like serum cystatin C and urinary NGAL. Notably, our study revealed significantly higher mean cystatin C levels in AKI patients, both preoperatively and at six hours postoperatively, highlighting its potential as an early AKI biomarker, consistent with previous studies [16,17]. Furthermore, all patients with AKI had elevated NGAL levels two hours postoperatively, consistent with numerous studies validating NGAL as an early and sensitive AKI biomarker in pediatric cardiac surgery [18].

Interestingly, we found a noteworthy association between lower intraoperative hematocrit levels (<30%) and a higher incidence of AKI, likely due to hemodilution or blood loss during surgery leading to renal hypoperfusion, consistent with prior research [19,20]. Our study also suggests the preemptive use of low-dose vasopressin infusion following the induction of anesthesia. Vasopressin, through its selective vasoconstrictive properties (efferent more than afferent), can help maintain adequate blood pressure and organ perfusion during surgery, potentially preserving renal function, increasing the GFR, and reducing the risk of CS-AKI [21,22].

Early blood transfusion at a rate of 4 ml/kg/hour was initiated to mitigate blood loss during critical steps such as sternotomy, cannulation of major vessels, and when the patient is placed on a cardiopulmonary bypass pump. This approach helps prevent intraoperative hypotension and preserves hematocrit levels.

The incidence of AKI in our study is notably lower than that reported in several prior studies that did not implement preemptive vasopressin or transfusion strategies [5,7,12,13]. To gain a clearer understanding of the effects of these interventions, we compared our results with the studies that did not utilize them. Table 7 presents a summary of AKI incidence rates in these studies, emphasizing the potential advantages of employing preemptive measures.

**Table 7:** Comparison of AKI incidence and use of preemptive interventions AKI: acute kidney injury

Study	Incidence of AKI (%)	Use of Vasopressin	Use of Blood Transfusion
Current Study	31.8	Yes	Yes
Li et al. [5]	42	No	Not mentioned
Alabbas et al. [23]	62	No	Not mentioned
Piggott et al. [24]	45	No	Not mentioned
Park et al. [13]	41.8	No	Not mentioned

Overall, our findings underscore the significance of perioperative hematocrit management and implementing strategies such as preemptive vasopressin infusion and maintaining hematocrit levels within a targeted range (>35%) [25-28]. These measures could prove pivotal in reducing the incidence of AKI and improving patient outcomes in this high-risk population, corresponding to the lower incidence rate of AKI in this study when compared to other similar studies. Our study highlights the need for heightened vigilance and proactive strategies to address the substantial burden of AKI in pediatric cardiac surgery patients.

Limitations

This study has a few limitations, including its small sample size and being conducted at a single center, which reduces the generalizability of the results to other institutions with different protocols or patient demographics. The observational nature, lacking both randomization and a control group, introduces potential selection bias. The duration of cardiopulmonary bypass is a variable factor that can significantly influence outcomes, serving as a potential confounding factor in the study. Additionally, the focus on short-term outcomes may miss the long-term impacts of AKI. Finally, while biomarkers such as NGAL and cystatin C are promising for early AKI detection, their routine use in clinical settings is not yet widespread, limiting the broader applicability of the findings.

## Conclusions

The present prospective observational study provides valuable insights into the incidence and risk factors associated with AKI in pediatric patients undergoing cardiac surgery. Our findings underscore the significant burden of AKI in this vulnerable patient population, with an incidence of 31.8%, and highlight the potential utility of novel biomarkers such as cystatin C and NGAL, as well as clinical parameters like intraoperative hematocrit levels, for early risk stratification and management. Implementing strategies like preemptive low-dose vasopressin infusion and maintaining optimal hematocrit levels during cardiac surgery may help mitigate the risk of AKI by preserving renal perfusion and function. These findings contribute to a better understanding of the modifiable risk factors associated with AKI in this high-risk population and may inform clinical strategies for risk mitigation and perioperative management, ultimately improving patient outcomes. To establish a definitive protocol, larger randomized studies are necessary. Additionally, future trials should aim to evaluate the effectiveness of preemptive low-dose vasopressin in improving renal perfusion, using the renal resistive index as the measurement tool.

## References

[1] Chesney RW, Kaplan BS (1975). Acute renal failure: an important complication of cardiac surgery in infants. J Pediatr.

[2] Rigden SP, Barratt TM, Dillon MJ, De Leval M, Stark J (1982). Acute renal failure complicating cardiopulmonary bypass surgery. Arch Dis Child.

[3] Sethi SK, Kumar M, Sharma R, Bazaz S, Kher V (2015). Acute kidney injury in children after cardiopulmonary bypass: risk factors and outcome. Indian Pediatr.

[4] Meersch M, Schmidt C, Van Aken H (2014). Validation of cell-cycle arrest biomarkers for acute kidney injury after pediatric cardiac surgery. PLoS One.

[5] Li S, Krawczeski CD, Zappitelli M (2011). Incidence, risk factors, and outcomes of acute kidney injury after pediatric cardiac surgery: a prospective multicenter study. Crit Care Med.

[6] Blinder JJ, Goldstein SL, Lee VV, Baycroft A, Fraser CD, Nelson D, Jefferies JL (2012). Congenital heart surgery in infants: effects of acute kidney injury on outcomes. J Thorac Cardiovasc Surg.

[7] Morgan CJ, Zappitelli M, Robertson CM (2013). Risk factors for and outcomes of acute kidney injury in neonates undergoing complex cardiac surgery. J Pediatr.

[8] Ji B, Undar A (2007). Comparison of perfusion modes on microcirculation during acute and chronic cardiac support: is there a difference?. Perfusion.

[9] Haase M, Bellomo R, Haase-Fielitz A (2010). Novel biomarkers, oxidative stress, and the role of labile iron toxicity in cardiopulmonary bypass-associated acute kidney injury. J Am Coll Cardiol.

[10] Zhang WR, Garg AX, Coca SG (2015). Plasma IL-6 and IL-10 concentrations predict AKI and long-term mortality in adults after cardiac surgery. J Am Soc Nephrol.

[11] Tóth R, Breuer T, Cserép Z (2012). Acute kidney injury is associated with higher morbidity and resource utilization in pediatric patients undergoing heart surgery. Ann Thorac Surg.

[12] Aydin SI, Seiden HS, Blaufox AD, Parnell VA, Choudhury T, Punnoose A, Schneider J (2012). Acute kidney injury after surgery for congenital heart disease. Ann Thorac Surg.

[13] Park SK, Hur M, Kim E (2016). Risk factors for acute kidney injury after congenital cardiac surgery in infants and children: a retrospective observational study. PLoS One.

[14] Lin CY, Chen YC (2012). Acute kidney injury classification: AKIN and RIFLE criteria in critical patients. World J Crit Care Med.

[15] Ronco C, Haapio M, House AA, Anavekar N, Bellomo R (2008). Cardiorenal syndrome. J Am Coll Cardiol.

[16] Zappitelli M, Bernier PL, Saczkowski RS (2009). A small post-operative rise in serum creatinine predicts acute kidney injury in children undergoing cardiac surgery. Kidney Int.

[17] Lassnigg A, Schmid ER, Hiesmayr M, Falk C, Druml W, Bauer P, Schmidlin D (2008). Impact of minimal increases in serum creatinine on outcome in patients after cardiothoracic surgery: do we have to revise current definitions of acute renal failure?. Crit Care Med.

[18] Forrest P (2001). Vasopressin and shock. Anaesth Intensive Care.

[19] Bilezikjian LM, Vale WW (1987). Regulation of ACTH secretion from corticotrophs: the interaction of vasopressin and CRF. Ann N Y Acad Sci.

[20] Singh VK, Sharma R, Agrawal A, Varma A (2009). Vasopressin in the pediatric cardiac intensive care unit: myth or reality. Ann Pediatr Cardiol.

[21] Zappitelli M, Krawczeski CD, Devarajan P (2011). Early postoperative serum cystatin C predicts severe acute kidney injury following pediatric cardiac surgery. Kidney Int.

[22] Meyer S, Gottschling S (2008). Effects of vasopressin on renal function in children with severe forms of shock. Crit Care Med.

[23] Alabbas A, Campbell A, Skippen P, Human D, Matsell D, Mammen C (2013). Epidemiology of cardiac surgery-associated acute kidney injury in neonates: a retrospective study. Pediatr Nephrol.

[24] Piggott KD, Soni M, Decampli WM (2015). Acute kidney injury and fluid overload in neonates following surgery for congenital heart disease. World J Pediatr Congenit Heart Surg.

[25] Kumar A, Ghotra GS, Raj S, Tiwari N, Ramamurthy HR (2023). Low-Dose vasopressin and renal perfusion in pediatric cardiac surgery. Ann Card Anaesth.

[26] Ricci Z, Netto R, Garisto C, Iacoella C, Favia I, Cogo P (2012). Whole blood assessment of neutrophil gelatinase-associated lipocalin versus pediatricRIFLE for acute kidney injury diagnosis and prognosis after pediatric cardiac surgery: cross-sectional study*. Pediatr Crit Care Med.

[27] Cantinotti M, Storti S, Lorenzoni V (2012). The combined use of neutrophil gelatinase-associated lipocalin and brain natriuretic peptide improves risk stratification in pediatric cardiac surgery. Clin Chem Lab Med.

[28] Riley AA, Jefferies JL, Nelson DP (2014). Peritoneal dialysis does not adversely affect kidney function recovery after congenital heart surgery. Int J Artif Organs.

